# Transcriptomics and Epigenomics in head and neck cancer: available repositories and molecular signatures

**DOI:** 10.1186/s41199-020-0047-y

**Published:** 2020-01-21

**Authors:** Mara S. Serafini, Laura Lopez-Perez, Giuseppe Fico, Lisa Licitra, Loris De Cecco, Carlo Resteghini

**Affiliations:** 1 Integrated Biology Platform, Department of Applied Research and Technology Development, Fondazione IRCCS Istituto Nazionale dei Tumori di Milano, Milan, Italy; 2 Life Supporting Technologies, Universidad Politécnica de Madrid, Madrid, Spain; 3 Head and Neck Medical Oncology Department, Fondazione IRCCS Istituto Nazionale dei Tumori di Milano, Milan, Italy; 4 University of Milan, Milan, Italy

**Keywords:** Head and neck squamous cell carcinoma, HNSCC, Transcriptomics, Epigenomics, Oral cavity, Oropharynx, Larynx, Hypopharynx, Prognosis, HPV

## Abstract

For many years, head and neck squamous cell carcinoma (HNSCC) has been considered as a single entity. However, in the last decades HNSCC complexity and heterogeneity have been recognized. In parallel, high-throughput *omics* techniques had allowed picturing a larger spectrum of the behavior and characteristics of molecules in cancer and a large set of omics web-based tools and informative repository databases have been developed. The objective of the present review is to provide an overview on biological, prognostic and predictive molecular signatures in HNSCC. To contextualize the selected data, our literature survey includes a short summary of the main characteristics of omics data repositories and web-tools for data analyses. The timeframe of our analysis was fixed, encompassing papers published between January 2015 and January 2019. From more than 1000 papers evaluated, 61 omics studies were selected: 33 investigating mRNA signatures, 11 and 13 related to miRNA and other non-coding-RNA signatures and 4 analyzing DNA methylation signatures. More than half of identified signatures (36) had a prognostic value but only in 10 studies selection of a specific anatomical sub-site (8 oral cavity, 1 oropharynx and 1 both oral cavity and oropharynx) was performed. Noteworthy, although the sample size included in many studies was limited, about one-half of the retrieved studies reported an external validation on independent dataset(s), strengthening the relevance of the obtained data. Finally, we highlighted the development and exploitation of three gene-expression signatures, whose clinical impact on prognosis/prediction of treatment response could be high. Based on this overview on omics*-related* literature in HNSCC, we identified some limits and strengths. The major limits are represented by the low number of signatures associated to DNA methylation and to non-coding RNA (miRNA, lncRNA and piRNAs) and the availability of a single dataset with multiple omics on more than 500 HNSCC (i.e. TCGA). The major strengths rely on the integration of multiple datasets through meta-analysis approaches and on the growing integration among *omics* data obtained on the same cohort of patients. Moreover, new approaches based on artificial intelligence and informatic analyses are expected to be available in the next future.

## Background

Head and neck squamous cell carcinoma (HNSCC) is the seventh most frequent cancer, with a worldwide incidence of 0.7 million new cases per year, and a low 5-year survival rate for both localized and advanced disease (69 and 34%, respectively) [1]. For several years, HNSCC has been considered as a single entity, since all sub-sites (i.e. oral cavity, oropharynx, larynx, hypopharynx) share a common epithelial precursor. Based on this assumption, treatment and biological analyses were mostly applied with no distinction for each of the sub-sites. However, clinical-pathological features and molecular changes, driving carcinogenesis [2], have helped in recognizing HNSCC complexity and heterogeneity. In addition, The Human Genome Project in 2003 [3] and following developments of next-generation sequencing (NGS) technologies have generated a cascade of high-throughput methodologies, altogether named *omics. Omics* have substantially led biology understanding to a deeper level for several cancer types, including HNSCC. In the present paper, we reviewed the main *omics* methodologies and the available resources for retrieving and analyzing omics data. Additionally, we updated our previous work [4] with the most recent published data in the context of HNSCC *Transcriptomics* and *Epigenomics,* considering these reviews as a continuum. The objective of the present work is to comprehensively review available information on transcriptomics and epigenomics in HNSCC to provide an overview on biological, prognostic and predictive molecular signatures.

### Main Omics methodologies

Biology is the result of the presence, expression, interaction, and regulation of different types of molecules. For their ability to account such a complexity, *omics* technologies have grown over the last two decades and they are now highly intertwined with other biological functional analysis [5]. Considering the classical cellular workflow of transcription (from DNA to mRNA) and translation (from mRNA to protein), *omics* can be presented as follows: i) *Genomics* has been introduced as the first high-throughput omics technique that impacted several aspects of clinical activity. It analyses the whole sequences of coding and non-coding portions of the genome, and targeted sequences (such as exome or clinical exome sequences). *Genomics* allows the identification of possibly relevant variants, such as single nucleotide polymorphisms (SNPs), copy number variation (CNV), mutations and translocations; ii) *Transcriptomics* involves all the RNA transcripts (with a particular attention in the last decade to mRNA, and more recently to long non-coding RNA [lncRNA]), monitor their differences in expression and infer the impacts of their alteration; iii) *Epigenomics* essentially studies DNA methylation variations and the functional consequences of the spatial behavior of the DNA (see also Table 1). Moreover, other cellular molecules have been analyzed by high-throughput methodologies and entered in the omics sciences, such as proteins, metabolites in general and lipids in particular (*Proteomics, Metabolomics, Lipidomics*). Recently, the omics suffix was also applied to emerging non-molecular fields: ‘radiomics’, the high-throughput mining of quantitative image features from clinically used medical imaging [6] and ‘metagenomics’, the assessment of the microbial communities inhabitant of the human body. More details about the characteristics of these other omics areas and methods are available elsewhere [7].

**Table 1:** The main *omics* techniques and their characteristics: the biological material analyzed, the major methodologies applied and the type of information achievable with them

Omics name	Material analyzed	Methodologies	Type of information
Genomics	DNA	Whole-genome sequencing	Complete genome sequence for identification of coding and non-coding sequence variants
		Exome sequencing or clinical exome-sequencing	Sequencing of protein-coding regions of the genome or genes known to be associated to a clinical phenotype
		Comparative genomic hybridization (aCGH)	Analysis of copy number alterations in the genome (comparison with a reference genome/sample)
Epigenomics/Transcriptomics	DNA/RNA	Whole or targeted DNA methylation	Identification of disfunctions in regulatory elements
		Whole or targeted miRNA	Sequencing or detection of differential expression of miRNA
		Whole or targeted long noncoding RNA	Sequencing or detection of differential expression of long noncoding RNA
		Whole or targeted mRNA	Sequencing or detection of differential expression of coding genes

### Available resources for retrieving and analyzing Omics data

The application of high-throughput techniques requires high computational capacity and expertise in handling large amounts of data. Consequently, repositories for omics have been created worldwide (Tables 2, 3). Most of these repositories are publicly accessible and useful for data consulting. The ArrayExpress archive is one of the ELIXIR Core Data Resources and stores data derived from array- and sequence-based experiments. Researchers can upload data if the provided content is compliant with the Minimum Information About a Microarray Experiment (MIAME) and the Minimum Information About a Next-generation Sequencing Experiment (MINSEQE) standards [8]. ArrayExpress experiment results are available as: i) metadata information with the experiment description, protocol procedures, sample annotations and author information; ii) raw experiment data; iii) processed data. ArrayExpress enables access to BioSamples [9], another ELIXIR repository, providing a store to collect metadata, about biological samples. Gene Expression Omnibus (GEO) is a public repository supported by the National Cancer Center for Biotechnology Information (NCBI) and it archives MIAME- and MINSEQE-compliant functional genomics data of all organisms. Data derived from array- and sequence-based analyses are available, comprising dataset information, experiment variable subsets, expression value measurements, gene symbols and, comprehensive gene annotation. Additionally, GEO offers several functionalities for data analysis through GEO DataSet database, such as gene search, comparison of samples sets, inspection of cluster heat-maps, execution of experimental design and value distribution with box plot visualization support. Another available repository is The Cancer Genome Atlas (TCGA), which contains only human cancer data and, for this reason, differs from the previously described repository. TCGA was born as collaboration between the National Cancer Institute (NCI) and the National Human Genome Research Institute (NHGRI) and was upgraded and merged with the Pan-Cancer Atlas [10]. Both TCGA and Pan-Cancer Atlas offer a reclassification of human tumor types based on molecular similarity, a molecular landscape of the oncogenic processes and a comprehensive analysis of tumor signaling pathways. Only TCGA and Pan-Cancer consortium members have the access to submit omics data and data upload is continuously in progress. TCGA dataset system contains 25 human cancer types and it is provided free of charge. Its exploration is supported by descriptive charts. A controlled access is required for data downloading. Another important repository is the Functional Annotation of the Mammalian Genome (FANTOM), an international research consortium that encompasses the field of transcriptome analyses. The project delivered the FANTOM5 collection, a data series supporting structure of mammalian transcriptome atlases in diverse cell types. FANTOM5 data contains: Cap Analysis of Gene Expression (CAGE) and annotation tables; pathway enrichment and co-expression cluster analysis; enhancers; results of *de-novo* and motif activity analysis; sample ontology and ontology term enrichment; CAGE peaks identified by specific classifier and visualization tools.

**Table 2:** Main public repositories and their features

SOURCE	ArrayExpress	GEO (Gene expression omnibus) - NCBI (The National Center for Biotechnology Information)	TCGA (The Cancer Genome Atlas)	FANTOM5
Link	www.ebi.ac.uk/arrayexpress	www.ncbi.nlm.nih.gov/geo/summary	portal.gdc.cancer.gov	fantom.gsc.riken.jp
Repository data	Public archive of Genomics/Functional Genomics Data all organisms, normal and disease associated	Public archive of Functional Genomics Data of all organisms, normal and disease associated	Public dataset of Omics Data of Human Cancer	International consortium of functional annotations of mammalian coding and non coding portion of genome
Submitting authors	All the researchers, following the MIAME and MINSEQE rules	All the researchers, following the MIAME and MINSEQE rules	Only TCGA/Pancancer consortium members	Only FANTOM5 consortium members
Access for downloading	Free	Free	Required for downloading controlled-access data	Free
Type of available data	Array- and sequence-based data. Meta-analysis not accepted. Updated daily	Array- and sequence-based data. Meta-analysis not accepted. Updated daily	Omics data generated by TCGA Consortium and Pancancer Atlas. Continuously in progress	Transcriptomics data. Updated monthly
Query types	See Elixir Core Data Resource in Table 3	Download analysis (find genes, compare 2 sets of samples, cluster heatmaps, experiment design and value distribution)	See tools for querying TCGA in Table 3	Visualization tools such as ZENBU, a system for data integration, analysis and visualization of NGS based data

**Table 3:** Details of the largest and most utilized web-tools for omics analyses and a list of free access repositories to retrieve useful information related to mRNA, microRNA and other non coding-RNA

SOURCE		link/access	Type of tool/repository	Type of available data
Elixir Core Data Resource	Ensembl	www.ensembl.org/index;	Genome browser that: annotates genes, computes multiple alignments, predicts regulatory function and collects disease data.	Vertebrates organisms data
		free access		
	European Genome-phenome Archive (EGA)	www.ebi.ac.uk/ega/;	Database containing all types of sequence and genotype experiments, including case-control, population, and family studies.	Human, normal and disease associated
		login requested		
	Rfam	rfam.xfam.org/;	Database of non-coding RNA families	Vertebrates organisms
		free access		
	RNAcentral	rnacentral.org/;	Web tool with access to a set of non-coding RNA sequences, allowing: sequence search, public Postgres database and genome browser. It enables integrated text search, sequence similarity search, bulk downloads, and programmatic data access.	Vertebrates and other organisms
		free access		
Tools for querying TCGA	The cancer omics atlas (TCOA)	www.tcoa.cpu.edu.cn;	This tool provides useful functions complementary to other existing tools, for fast and straightforward querying of TCGA	Human cancer data
		free access		
	Broad Institute	http://gdac.broadinstitute.org/;	Web tool to systematize analysis from TCGA, with the following functionalities: gene expression Viewer, data analysis (overview, results, methods and data), and data download.	Human cancer data
		Required TCGA controlled-access data		
	OncoLnc	http://www.oncolnc.org/;	Web tool analyzes TCGA in terms of survival by selecting the target genes.	Human cancer data
		free access to survival data (raw data not accessible)		
	TCGA Batch Effects Viewer	http://bioinformatics.mdanderson.org/tcgambatch/	Webtool designed to to help assess, diagnose and correct for any batch effects in TCGA data.	Human cancer data
	cBioPortal	http://www.cbioportal.org; Open source license via GitHub	Software allowing integrative analysis of cancer genomics	Human cancer data

In parallel, a large set of omics web-based tools and an increasing amount of informative repository databases have been developed (Table 3). ELIXIR [11] is an intergovernmental organization, composed by 23 members and over 180 research organizations among Europe. It is a Core Data resource with several web-based bioinformatics tools such as: i) Ensembl*,* a browser for DNA sequences and assemblies, provided by international projects on vertebrate genomes that accommodates annotated genes, computes multiple alignments, predicts regulatory function and collects disease data; ii) European Genome-phenome Archive (EGA)*,* a web-tool, providing information from genetic and phenotypic data coming from biomedical research projects; iii) Rfam, a database collecting multiple sequence alignments, consensus secondary structures and covariance models (CMs) for non-coding RNA families; and iv) RNAcentral, provided by collaborating groups (ENA, Ensembl, GENCODE, miRBase), bringing integrated access to a comprehensive and up-to-date set of non-coding RNA sequences. Furthermore, a number of web-based tools or software querying TCGA are available: i) The Cancer Omics Atlas (TCOA), providing useful functions complementary to other existing tools, such as querying of gene, miRNA and protein expression, somatic mutations (based on a single molecule or cancer type correlations of gene–gene, miRNA–miRNA, protein–protein, gene–miRNA and gene–protein), and their correlation with survival prognosis in cancer patients; ii) Broad Institute, allowing systematic analysis on TCGA data and comparison with other diseases; iii) OncoLnc*,* analyzing patients survival (Kaplan-Meier curves) according to mRNA, miRNA, lncRNA expression levels; and iv) TCGA Batch Effects Viewer [12], a tool specifically designed to diagnose and correct for any batch effects in TCGA data; v) cBioPortal [13], a software allowing genomic analysis both from population or a single patient of multiple cancer types. In addition to these two main types of resources (i.e. ELIXIR and TCGA querying tools), it should be mentioned: i) MiTranscriptome [14], a catalog of human long poly-adenylated RNA transcripts, from samples encompassing different cancer and tissue types; ii) KM plotter [15], a tool assessing the effect of genes/miRNA on overall survival data for biomarker discovery; iii) Bioconductor*,* an open-source tool based on R programming language for the analysis and comprehension of high-throughput data and enabling generation of workflows for multiple data types, data preprocessing, statistical inference, regression, network analysis, machine learning, multiomics integration and visualization. For further information about other tools, databases and websites also see the following reviews [16–18].

### Strategy of search and selection of studies

Literature surveys of HNSCC genomics [2] and proteomics [19] have already been conducted and published in the past years. For this reason, we decided to focus on HNSCC transcriptomics and epigenomics studies, characterizing signatures related to biology, prognosis and prediction of treatment response. The timeframe of our analysis was fixed, encompassing papers published between January 2015 and January 2019. The purpose of this choice was to partially overlap with a previous review on transcriptomics data [4] and was dictated by the evidence that both epigenomics (DNA methylation) and transcriptomics based on non-coding RNA (miRNA, lncRNA and piRNAs) are advancing and growing only in recent years. A web-based search has been performed in the following databases: Pubmed, ArrayExpress and GEO. The combination of the following keywords has been used: “gene expression” or “methylation” or “miRNA” or “transcriptomics” or “sequencing” or “microarray” AND “head and neck cancer” or “HNSCC”. The title and the abstract of all potentially relevant studies were assessed for their contents before the retrieval of full articles. The full text of each selected study was carefully evaluated. Eligible studies were required to meet the following inclusion criteria: publication which data has been obtained using HNSCC tumor tissue; the number of cases per each analysis had to be ≥40. Moreover, the following exclusion criteria were applied: non-English publication; case reports, letters and reviews; expression studies of individual preselected candidate gene/miRNA/DNA methylation site; and data obtained on not human samples/cell lines/different patient materials (serum, plasma, saliva). Papers that fulfilled the inclusion criteria were processed for data.

### Analysis of recent Epigenomics and Transcriptomics data

Over more than 1000 papers have been analyzed from which we retrieved a total of 61 omics studies fulfilling inclusion and exclusion criteria [20–80].

The selected studies are listed in the Additional file 1: Table S1, subdivided according to the analyzed feature, such as mRNA, miRNA, non-coding RNAs and DNA methylation, respectively. This subdivision was univocal for 54 studies. Conversely, authors of 6 studies analyzed more than one feature and it was decided to classify them on the basis of the feature with higher relevance, according to paper aim. Data in the supplementary tables are reported as: i) the name of the identified signature according to the authors of publication; ii) the type of feature analyzed; iii) the information provided by the signature (biology, prognosis, prediction of treatment response); iv) selection based on anatomical site (oral cavity, oropharynx, larynx, hypopharynx) or HPV-status for data analysis; v) the ID of the dataset; and vi) availability of independent validation.

**Fig. 1:**
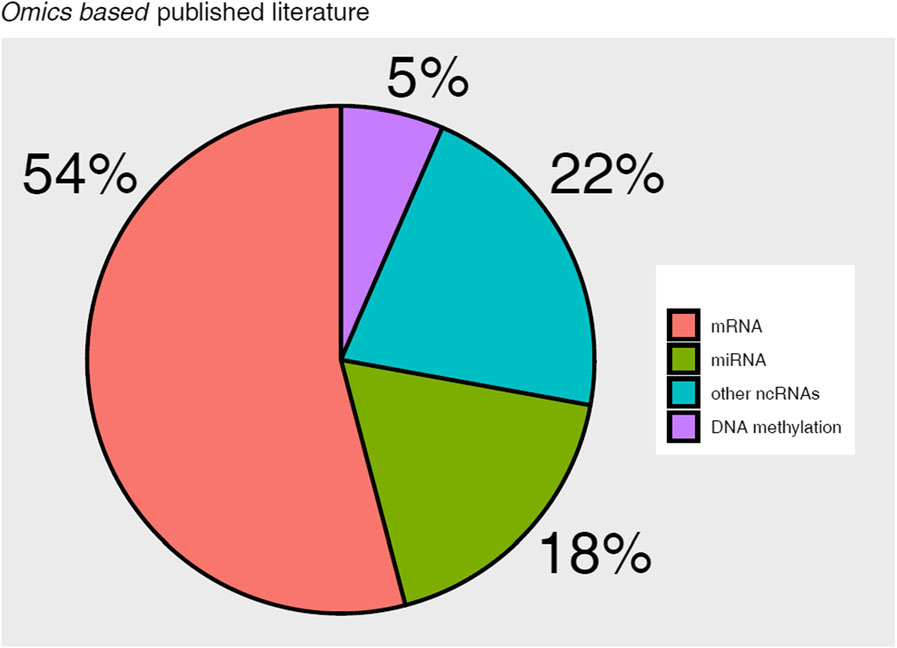
Omics based published literature. Visual distribution (%) of the retrieved 61 published papers, according to the studied feature

**Fig. 2:**
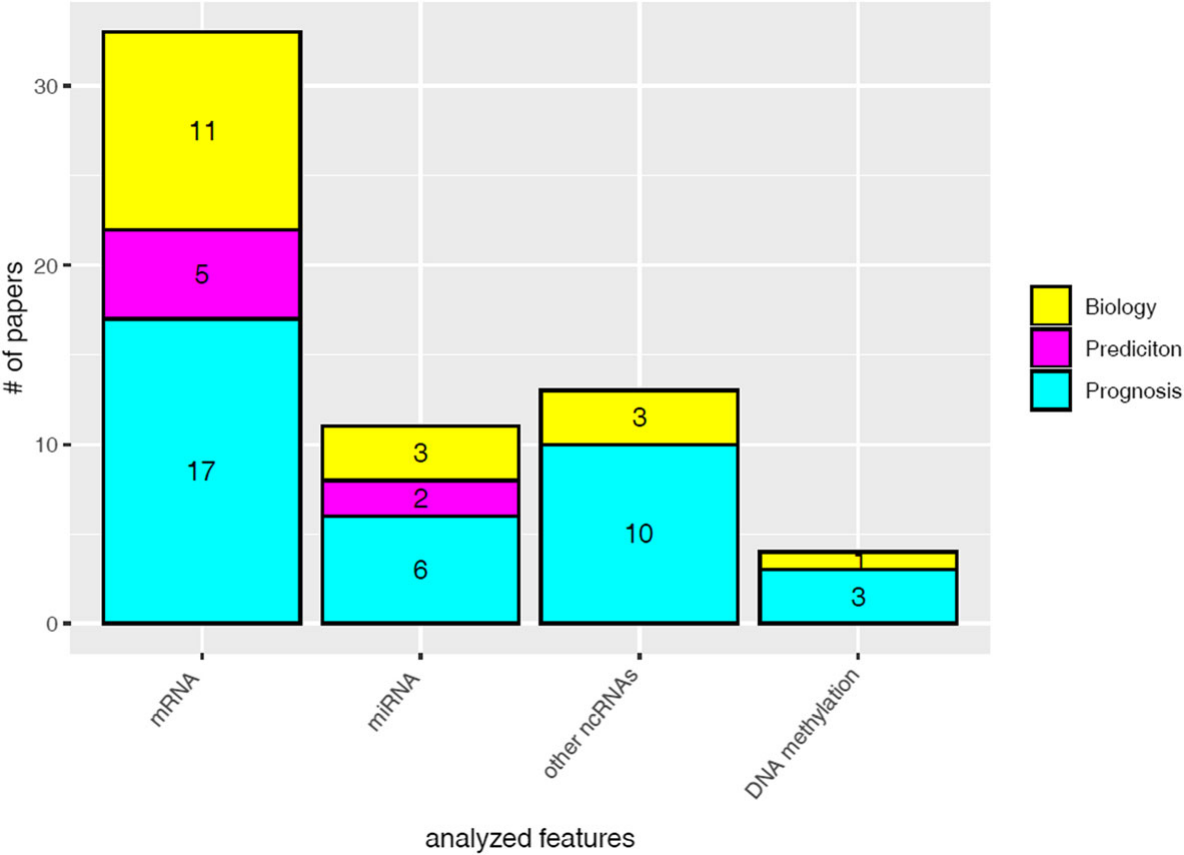
Comparison of each studied feature, according to their analysis objective (biology, prognosis, prediction of treatment response)

A large portion of the studies regarded mRNA signatures (33/61) [19–51], while a minor portion of the studies considered miRNA signatures (11/61) [52–62] and other non-coding RNA signatures (13/61) [ 63–76]. Only 4/61 [77–80] studies identified signatures by a high-throughput omics analysis of methylated DNA. The percentage distribution of the studies according to the type of features analyzed is reported in Fig. 1. The information provided by the signature, i.e. biology, prognosis and prediction of treatment response, are depicted in Fig. 2.

Most of the identified signatures had a prognostic value (36/61); on the contrary, only a minority (7/61) was related to prediction of treatment response. No signature derived from the study of other non-coding RNAs or DNA methylation had prognostic or predictive value.

A total of 21 studies selected HNSCC samples based on specific anatomical site or HPV status, while no selection was applied in the remaining 40 studies. Another remarkable aspect, regarding datasets of the analyzed studies, is the presence or absence of internal validation in the same publication: only half of the studies included in our analyses performed a validation in independent datasets.

Noteworthy, the majority of transcriptomic and epigenomic datasets used to define or validate the signature under evaluation were generated by TCGA. TCGA datasets were the only ones included in meta-analyses or validation set in 46/61 studies: mRNA (24/33); miRNA expression (7/11); DNA methylation (3/4); and 12/13 studies on non-coding RNA.

Even though all the selected studies deserve attention, a detailed analysis of each one is out of scope of this survey. However, we decided to comment the development and exploitation of three gene-expression signatures, whose clinical impact on prognosis/prediction of treatment response could be high. Two signatures were initially identified by analysis of HNSCC cell lines. These gene classifiers/indexes were subsequently tested in historical retrospective clinical cohorts and validated in prospective clinical studies, showing promising prognostic or predictive ability. The first signature is the radio-sensitivity index (RSI), whose development and clinical validation in three data sets of rectal, esophageal and HNSCC was originally described in 2009 [81]. The RSI was further commented in 2017 [82] and constituted the pillar for proposing a genome-based model for adjusting radiotherapy dose (GARD) as it was applied to a large retrospective, cohort-based study [83]. At present, a company (Precision Genomic Radiation Therapy platform: pGRT™) developed a mathematical approach to the integration of genomics into radiation treatment and planning; this application is central to the Cvergenx patented RSI and GARD (https://www.cvergenx.com/). At the present, this nomogram is in development for clinical purposes in other tumor types, but not in HNSCC.

The second signature is the 15-gene hypoxia classifier, first described between 2010 and 2011 [84, 85] and then validated as prognostic signature in retrospective series [86]. A patent application is currently pending on this method for determination of clinically relevant hypoxia in cancer specimen (WO/2012/146259). The clinical relevance of this signature is highlighted by the decision to conduct a double blind randomized multicenter phase III study, the Intergroup EORTC-1219-ROG-HNCG/DAHANCA-29 trial (NCT01880359). This study is designed to prospectively evaluate if nimorazole, a radio-sensitizer, can improve the effect of accelerated concomitant chemo-radiotherapy with cisplatin on the locoregional control rate in patients with newly diagnosed HPV 16 negative stage III-IV carcinoma of the larynx, oropharynx or hypopharynx. The study is designed to stratify patients according to the 15-gene signature in order to determine if the treatment benefit is larger in patients who carry a hypoxia- profile.

The third signature [32], published in 2016 by some of the Authors of the present paper, analysed HNSCC gene expression of patients with short and prolonged responses to cetuximab- and platinum-based chemotherapy. Basal subtype traits, including signatures of EGFR signalling and hypoxic differentiation, characterized patients with long response whereas short-response patients showed RAS activation. These results were commented upon [87] as an important step in the identification of candidate predictive biomarkers of response to cetuximab–platinum therapy in recurrent/metastatic HNSCC patients. Furthermore, the predictive power of the signature were refined by the creation of a common network with results from miRNA analyses of the same sample set [58]. At last, a validation was successfully completed in different sample sets of recurrent/metastatic HNSCC treated with different anti-EGFR agent, specifically the phase II PANI01 trial employing panitumumab [88] and a window of opportunity trial evaluating pre-operative afatinib [89]. Despite differences in clinical settings and anti-EGFR inhibitor treatments, prediction of response by the previously identified Cluster 3 signature and selected miRNAs was comparable. Cluster 3 signature is characterized, beside hypoxia, by others functional pathways including altered metabolism.

## Conclusions and future directions

In the last decade, several progresses have been achieved not only in the methodology for “omics” analyses but also in availability of data repositories and web-based tools for the storage and the analysis of the enormous amount of data generated. Despite these progresses, the present literature revision highlighted that most of published works on HNSCC are not omics-based. In fact, we were able to retrieve only 61 such studies out of more than 1000 that were initially identified in our research.

Based on this overview on omics*-related* literature in HNSCC, we identified three major limits: i) the classical epigenomics area (DNA methylation) and the omics based on non-coding RNA (miRNA, lncRNA and piRNAs) have been analyzed quite recently; ii) the limited sample size included in most “omics” studies; iii) the largest dataset for HNSCC at present available is TCGA; and iv) even if there was an evident expansion of omics-related HNSCC publications starting from 2017, this increase should be partially attributed to the reiterated bioinformatic analysis of the TCGA dataset. To overcome the issues, the integration of multiple datasets through a meta-analysis approach has been reported to offer advantages, improving the reliability of results [21, 52]. However, some important aspects included in the more recent analyses should be highlighted. The presence of HPV infection in HNSCC, especially those arising from the oropharynx, has a well-known and profound impact on prognosis. The recently released 8th edition of the American Joint Committee on Cancer (AJCC) staging system has introduced major differences in oropharynx squamous cell carcinoma, now staged according to p16 status [90]. Few recent *omic*-based analyses dissected the biologic aspect underling this phenomenon [91] and very recent data indicate a clear association between subtypes and different prognosis [52]. A deeper knowledge of molecular biology and mechanisms of carcinogenesis in HPV-related HNSCC will be critical in order to further differentiate patient’s prognosis and therefore improve disease management. Of note, in the context of growing epidemics [92] the identification of ideal candidate for safe de-escalated therapy should be focused on genomic and molecular factors in order to achieve a successful application of the precision medicine ideal [93]. Another point of emphasis derived from the present overview is the growing integration among *omics* data obtained on the same cohort of patients. Even if these experiences are still limited for HNSCC and in terms of types of *omics* employed and amount of published studies [23, 27, 43, 58, 66, 76], these initiatives enabling to better dissect cancer complexity deserve further investigations. Moreover, we can expect that new approaches based on artificial intelligence will be available in the next future dealing with more complex data even integrating multi-omics layers [94]. At present, a particular method, self-organizing maps (SOM)-machine learning offers a practical solution when hundreds of samples are profiled for thousands of genes as microarray/RNAseq and a number of studies on different cancer types proved its robustness [18, 19]. As an example SOM enabled to separate oropharynx p16 positive tumors in three clusters with different prognosis [52]. Future informatic analyses are expected to: i) identify and implement services to retrieve *omics* data from public repositories; ii) harmonize *omics* data in order to merge different data sources in one integrated, HNSCC-specific dataset; and iii) explore the resulting dataset with dedicated techniques. Finally, we have to acknowledge not only the important anatomical site specific contribute of TCGA on HNSCC [95], but also the recent contribution of the Cancer Genome Atlas Pan-Cancer analysis project, which, by a multiplatform analysis of different cancer types [96, 97], revealed a molecular classification within and across tissues of origin. In particular, the analyses of 12 and 42 different cancer types by Campbell et al. [97] and Chen et al. [28], respectively, enabled to reveal that: i) squamous cell cancers from different tissue sites may be distinguished from other cancers and may be subclassified molecularly by squamous cell pathways and programs providing candidates for therapy; and ii) a small subset of HNSCC expresses evident traits of neuro-endocrinicity. In addition to the Cancer Genome Atlas upcoming data, in the next years we hope to witness a surge of new *omics*-based analyses in HNSCC, and based on new, large and rigorously clinically annotated datasets. An example is represented by the European Commission funded project named “Big Data and Models for Personalized Head and Neck Cancer Decision Support (BD2Decide)” (ClinicalTrial.gov Identifier NCT02832102, http://www.bd2decide.eu/). The project, started on 2016 and expected to be concluded at the end of 2019, aims at the definition of a prognostic tool based on the integration of multi-*omics* analyses of a large dataset of locoregionally advanced HNSCC.

## Supplementary information

**Additional file 1.** Supplementary Table

## Data Availability

This is a review article and there is no raw data related to this manuscript for data sharing.

## Abbreviations

AJCC: American Joint Committee on Cancer
GEO: Gene Expression Omnibus
HNSCC: Head and neck squamous cell carcinoma
HPV: Human papilloma virus; GEO; TGCA; SOM; Elixir
SOM: Self-Organizing Map
TGCA: The Cancer Genome Atlas
