# Supplementary material for: Transcriptomics and Epigenomics in head and neck cancer: available repositories and molecular signatures
Source: Cancers Head Neck. 2020 Jan 21;5:2. doi: 10.1186/s41199-020-0047-y (PMC6971871; doi:10.1186/s41199-020-0047-y)
Supplement: Supplementary file 1 — Additional file 1. Supplementary Table [file 41199_2020_47_MOESM1_ESM.zip › References of Supplementary Table A1.docx]

20. Wichmann G, Rosolowski M, Krohn K, et al. The role of HPV RNA transcription, immune response-related gene expression and disruptive TP53 mutations in diagnostic and prognostic profiling of head and neck cancer . *Int J Cancer*. 2015;137(12):2846-2857. doi:10.1002/ijc.29649

21. De Cecco L, Nicolau M, Giannoccaro M, et al. Head and neck cancer subtypes with biological and clinical relevance: Meta-analysis of gene-expression data. *Oncotarget*. 2015;6(11):9627-9642. doi:10.18632/oncotarget.3301

22. Keck MK, Zuo Z, Khattri A, et al. Integrative analysis of head and neck cancer identifies two biologically distinct HPV and three non-HPV subtypes. *Clin Cancer Res*. 2015;21(4):870-881. doi:10.1158/1078-0432.CCR-14-2481

23. Zhang W, Edwards A, Fang Z, Flemington EK, Zhang K. Integrative Genomics and Transcriptomics Analysis Reveals Potential Mechanisms for Favorable Prognosis of Patients with HPV-Positive Head and Neck Carcinomas. *Sci Rep*. 2016;6(April):1-14. doi:10.1038/srep24927

24. Zhang Y, Koneva LA, Virani S, et al. Subtypes of HPV-Positive Head and Neck Cancers Are Associated with HPV Characteristics, Copy Number Alterations, PIK3CA Mutation, and Pathway Signatures. *Clin Cancer Res*. 2016;22(18):4735-4745. doi:10.1158/1078-0432.CCR-16-0323

25. Cao B, Wang Q, Zhang H, Zhu G, Lang J. Two immune-enhanced molecular subtypes differ in inflammation, checkpoint signaling and outcome of advanced head and neck squamous cell carcinoma. *Oncoimmunology*. 2018;7(2). doi:10.1080/2162402X.2017.1392427

26. Chen YP, Wang YQ, Lv JW, et al. Identification and validation of novel microenvironment-based immune molecular subgroups of head and neck squamous cell carcinoma: Implications for immunotherapy. *Ann Oncol*. 2019;30(1):68-75. doi:10.1093/annonc/mdy470

27. Costa RL, Boroni M, Soares MA. Distinct co-expression networks using multi-omic data reveal novel interventional targets in HPV-positive and negative head-and-neck squamous cell cancer. *Sci Rep*. 2018;8(1):1-13. doi:10.1038/s41598-018-33498-5

28. Chen F, Zhang Y, Gibbons DL, et al. Pan-cancer molecular classes transcending tumor lineage across 32 cancer types, multiple data platforms, and over 10,000 cases. *Clin Cancer Res*. 2018;24(9):2182-2193. doi:10.1158/1078-0432.CCR-17-3378

29. Irimie AI, Braicu C, Cojocneanu R, et al. Differential Effect of Smoking on Gene Expression in Head and Neck Cancer Patients. *Int J Environ Res Public Health*. 2018;15(7):1558. doi:10.3390/ijerph15071558

30. Saidak Z, Clatot F, Chatelain D, Galmiche A. A gene expression profile associated with perineural invasion identifies a subset of HNSCC at risk of post-surgical recurrence. *Oral Oncol*. 2018;86:53-60. doi:10.1016/j.oraloncology.2018.09.005

31. Sanati N, Iancu OD, Wu G, Jacobs JE, McWeeney SK. Network-Based Predictors of Progression in Head and Neck Squamous Cell Carcinoma. *Front Genet*. 2018;9:183. doi:10.3389/fgene.2018.00183

32. Bossi P, Bergamini C, Siano M, et al. Functional Genomics Uncover the Biology behind the Responsiveness of Head and Neck Squamous Cell Cancer Patients to Cetuximab. *Clin Cancer Res*. 2016;22(15):3961-3970. doi:10.1158/1078-0432.CCR-15-2547

33. Foy J-P, Bazire L, Ortiz-Cuaran S, et al. A 13-gene expression-based radioresistance score highlights the heterogeneity in the response to radiation therapy across HPV-negative HNSCC molecular subtypes. *BMC Med*. 2017;15(1):165. doi:10.1186/s12916-017-0929-y

34. Chen X, Yan B, Lou H, et al. Immunological network analysis in HPV associated head and neck squamous cancer and implications for disease prognosis. *Mol Immunol*. 2018;96(February):28-36. doi:10.1016/j.molimm.2018.02.005

35. Chen J, Fu G, Chen Y, Zhu G, Wang Z. Gene-expression signature predicts survival benefit from postoperative chemoradiotherapy in head and neck squamous cell carcinoma. *Oncol Lett*. 2018;16(2):2565-2578. doi:10.3892/ol.2018.8964

36. You GR, Cheng AJ, Lee LY, et al. Prognostic signature associated with radioresistance in head and neck cancer via transcriptomic and bioinformatic analyses. *BMC Cancer*. 2019;19(1):1-11. doi:10.1186/s12885-018-5243-3

37. Tawk B, Schwager C, Deffaa O, et al. Comparative analysis of transcriptomics based hypoxia signatures in head- and neck squamous cell carcinoma. *Radiother Oncol*. 2016;118(2):350-358. doi:10.1016/j.radonc.2015.11.027

38. Guo W, Chen X, Zhu L, Wang Q. A six-mRNA signature model for the prognosis of head and neck squamous cell carcinoma. *Oncotarget*. 2017;8(55):94528-94538. doi:10.18632/oncotarget.21786

39. Mes SW, Beest D te, Poli T, et al. Prognostic modeling of oral cancer by gene profiles and clinicopathological co-variables. *Oncotarget*. 2017;8(35):59312-59323. doi:10.18632/oncotarget.19576

40. Qiu Z, Sun W, Gao S, et al. A 16-gene signature predicting prognosis of patients with oral tongue squamous cell carcinoma. *PeerJ*. 2017;5:e4062. doi:10.7717/peerj.4062

41. Shen S, Bai J, Wei Y, et al. A seven-gene prognostic signature for rapid determination of head and neck squamous cell carcinoma survival. *Oncol Rep*. 2017;38(6):3403-3411. doi:10.3892/or.2017.6057

42. Yang B, Chen Z, Huang Y, Han G, Li W. Identification of potential biomarkers and analysis of prognostic values in head and neck squamous cell carcinoma by bioinformatics analysis. *Onco Targets Ther*. 2017;10:2315-2321. doi:10.2147/OTT.S135514

43. Zhang ZL, Zhao LJ, Chai L, et al. Seven LncRNA-mRNA based risk score predicts the survival of head and neck squamous cell carcinoma. *Sci Rep*. 2017;7(1):309. doi:10.1038/s41598-017-00252-2

44. Lee DJ, Eun Y-GG, Rho YS, et al. Three distinct genomic subtypes of head and neck squamous cell carcinoma associated with clinical outcomes. *Oral Oncol*. 2018;85(January):44-51. doi:10.1016/j.oraloncology.2018.08.009

45. Namani A, Matiur Rahaman M, Chen M, Tang X. Gene-expression signature regulated by the KEAP1-NRF2-CUL3 axis is associated with a poor prognosis in head and neck squamous cell cancer. *BMC Cancer*. 2018;18(1):46. doi:10.1186/s12885-017-3907-z

46. Schmidt S, Linge A, Zwanenburg A, et al. Development and Validation of a Gene Signature for Patients with Head and Neck Carcinomas Treated by Postoperative Radio(chemo)therapy. *Clin Cancer Res*. 2018;24(6):1364-1374. doi:10.1158/1078-0432.CCR-17-2345

47. Tian S, Meng G, Zhang W. A six-mRNA prognostic model to predict survival in head and neck squamous cell carcinoma. *Cancer Manag Res*. 2019;11:131-142. doi:10.2147/CMAR.S185875

48. Wintergerst L, Selmansberger M, Maihoefer C, et al. A prognostic mRNA expression signature of four 16q24.3 genes in radio(chemo)therapy-treated head and neck squamous cell carcinoma (HNSCC). *Mol Oncol*. 2018;12(12):2085-2101. doi:10.1002/1878-0261.12388

49. Xu X, Li M, Hu J, et al. Expression profile analysis identifies a two-gene signature for prediction of head and neck squamous cell carcinoma patient survival. *J Cancer Res Ther*. 2018;14(7):1525. doi:10.4103/jcrt.jcrt_557_18

50. Wang W, Lim WK, Leong HS, et al. An eleven gene molecular signature for extra-capsular spread in oral squamous cell carcinoma serves as a prognosticator of outcome in patients without nodal metastases. *Oral Oncol*. 2015;51(4):355-362. doi:10.1016/j.oraloncology.2014.12.012

51. Zhao X, Sun S, Zeng X, Cui L. Expression profiles analysis identifies a novel three-mRNA signature to predict overall survival in oral squamous cell carcinoma. *Am J Cancer Res*. 2018;8(3):450-461. http://www.ncbi.nlm.nih.gov/pubmed/29637000. Accessed July 24, 2019.

52. Locati LD, Serafini MS, Iannò MF, et al. Mining of self-organizing map gene-expression portraits reveals prognostic stratification of HPV-positive Head and Neck Squamous cell carcinoma. *Cancers (Basel)*. 2019;11(8):1057. doi:10.3390/cancers11081057

53. An F, Zhang Z, Xia M, Xing L. Subpath analysis of each subtype of head and neck cancer based on the regulatory relationship between miRNAs and biological pathways. *Oncol Rep*. 2015;34(4):1745-1754. doi:10.3892/or.2015.4150

54. Krishnan AR, Zheng H, Kwok JG, et al. A comprehensive study of smoking-specific microRNA alterations in head and neck squamous cell carcinoma. *Oral Oncol*. 2017;72:56-64. doi:10.1016/j.oraloncology.2017.07.009

55. Wong N, Khwaja SS, Baker CM, et al. Prognostic microRNA signatures derived from The Cancer Genome Atlas for head and neck squamous cell carcinomas. *Cancer Med*. 2016;5(7):1619-1628. doi:10.1002/cam4.718

56. Sass S, Pitea A, Unger K, Hess J, Mueller NS, Theis FJ. MicroRNA-target network inference and local network enrichment analysis identify two microrna clusters with distinct functions in head and neck squamous cell carcinoma. *Int J Mol Sci*. 2015;16(12):30204-30222. doi:10.3390/ijms161226230

57. Chen L, Wen Y, Zhang J, et al. Prediction of radiotherapy response with a 5-microRNA signature-based nomogram in head and neck squamous cell carcinoma. *Cancer Med*. 2018;7(3):726-735. doi:10.1002/cam4.1369

58. De Cecco L, Giannoccaro M, Marchesi E, et al. Integrative miRNA-Gene Expression Analysis Enables Refinement of Associated Biology and Prediction of Response to Cetuximab in Head and Neck Squamous Cell Cancer. *Genes (Basel)*. 2017;8(1). doi:10.3390/genes8010035

59. Citron F, Armenia J, Franchin G, et al. An integrated approach identifies mediators of local recurrence in head and neck squamous carcinoma. *Clin Cancer Res*. 2017;23(14):3769-3780. doi:10.1158/1078-0432.CCR-16-2814

60. Hess J, Unger K, Maihoefer C, et al. A five-microRNA signature predicts survival and disease control of patients with head and neck cancer negative for HPV infection. *Clin Cancer Res*. 2019;25(5):1505-1516. doi:10.1158/1078-0432.CCR-18-0776

61. Hui L, Wu H, Yang N, Guo X, Jang X. Identification of prognostic microRNA candidates for head and neck squamous cell carcinoma. *Oncol Rep*. 2016;35(6):3321-3330. doi:10.3892/or.2016.4698

62. Nunez Lopez YO, Victoria B, Golusinski P, Golusinski W, Masternak MM. Characteristic miRNA expression signature and random forest survival analysis identify potential cancer-driving miRNAs in a broad range of head and neck squamous cell carcinoma subtypes. *Reports Pract Oncol Radiother*. 2018;23(1):6-20. doi:10.1016/j.rpor.2017.10.003

63. Shi H, Chen J, Li Y, et al. Identification of a six microRNA signature as a novel potential prognostic biomarker in patients with head and neck squamous cell carcinoma. *Oncotarget*. 2016;7(16):21579-21590. doi:10.18632/oncotarget.7781

64. Nohata N, Abba MC, Gutkind JS. Unraveling the oral cancer lncRNAome: Identification of novel lncRNAs associated with malignant progression and HPV infection. *Oral Oncol*. 2016;59:58-66. doi:10.1016/j.oraloncology.2016.05.014

65. Zou AE, Zheng H, Saad MA, et al. The non-coding landscape of head and neck squamous cell carcinoma. *Oncotarget*. 2016;7(32). doi:10.18632/oncotarget.9979

66. Zhao G, Fu Y, Su Z, Wu R. How Long Non-Coding RNAs and MicroRNAs Mediate the Endogenous RNA Network of Head and Neck Squamous Cell Carcinoma: A Comprehensive Analysis. *Cell Physiol Biochem*. 2018;50(1):342-352. doi:10.1159/000494009

67. Cao W, Liu J nan, Liu Z, et al. A three-lncRNA signature derived from the Atlas of ncRNA in cancer (TANRIC) database predicts the survival of patients with head and neck squamous cell carcinoma. *Oral Oncol*. 2017;65:94-101. doi:10.1016/j.oraloncology.2016.12.017

68. Feng L, Houck JR, Lohavanichbutr P, Chen C. Transcriptome analysis reveals differentially expressed lncRNAs between oral squamous cell carcinoma and healthy oral mucosa. *Oncotarget*. 2017;8(19):31521-31531. doi:10.18632/oncotarget.16358

69. Firmino N, Martinez VD, Rowbotham DA, Enfield KSS, Bennewith KL, Lam WL. HPV status is associated with altered PIWI-interacting RNA expression pattern in head and neck cancer. *Oral Oncol*. 2016;55:43-48. doi:10.1016/j.oraloncology.2016.01.012

70. de Lena PG, Paz-Gallardo A, Paramio JM, García-Escudero R. Clusterization in head and neck squamous carcinomas based on lncRNA expression: molecular and clinical correlates. *Clin Epigenetics*. 2017;9(1):1-11. doi:10.1186/s13148-017-0334-6

71. Krishnan AR, Korrapati A, Zou AE, et al. HHS Public Access. 2018:68-75. doi:10.1016/j.oraloncology.2016.12.022.Smoking

72. Diao P, Song Y, Ge H, et al. Identification of 4-lncRNA prognostic signature in head and neck squamous cell carcinoma. *J Cell Biochem*. 2019;120(6):10010-10020. doi:10.1002/jcb.28284

73. Liu G, Zheng J, Zhuang L, et al. A Prognostic 5-lncRNA Expression Signature for Head and Neck Squamous Cell Carcinoma. *Sci Rep*. 2018;8(1):15250. doi:10.1038/s41598-018-33642-1

74. Wang P, Jin M, Sun C, et al. A three-lncRNA expression signature predicts survival in head and neck squamous cell carcinoma (HNSCC). *Biosci Rep*. 2018;38(6):BSR20181528. doi:10.1042/bsr20181528

75. Zhao C, Zou H, Wang J, Shen J, Liu H. A Three Long Noncoding RNA-Based Signature for Oral Squamous Cell Carcinoma Prognosis Prediction. *DNA Cell Biol*. 2018;37(11):888-895. doi:10.1089/dna.2018.4317

76. Fang XN, Yin M, Li H, et al. Comprehensive analysis of competitive endogenous RNAs network associated with head and neck squamous cell carcinoma. *Sci Rep*. 2018;8(1):1-13. doi:10.1038/s41598-018-28957-y

77. Degli Esposti D, Sklias A, Lima SC, et al. Unique DNA methylation signature in HPV-positive head and neck squamous cell carcinomas. *Genome Med*. 2017;9(1):33. doi:10.1186/s13073-017-0419-z

78. Shen S, Wang G, Shi Q, et al. Seven-CpG-based prognostic signature coupled with gene expression predicts survival of oral squamous cell carcinoma. *Clin Epigenetics*. 2017;9(1):88. doi:10.1186/s13148-017-0392-9

79. Chen Y, Hei N, Zhao J, et al. A two-CpG-based prognostic signature for oral squamous cell carcinoma overall survival. *J Cell Biochem*. 2019;120(6):9082-9090. doi:10.1002/jcb.28182

80. Brennan K, Koenig JL, Gentles AJ, Sunwoo JB, Gevaert O. Identification of an atypical etiological head and neck squamous carcinoma subtype featuring the CpG island methylator phenotype. *EBioMedicine*. 2017;17:223-236. doi:10.1016/j.ebiom.2017.02.025
